# Overview of the Mutational Landscape in Primary Myelofibrosis and Advances in Novel Therapeutics

**DOI:** 10.31557/APJCP.2019.20.6.1691

**Published:** 2019

**Authors:** Jaleed Ahmed Gilani, Muhammad Areeb Ashfaq, Armaghan-e-Rehman Armaghan-e-Rehman, Adnan Abdul Jabbar, Tariq Siddiqui, Maliha Khan

**Affiliations:** 1 *Ziauddin Hospital, *; 2 *Aga Khan University Hospital, *; 4 *Section of Oncology, Aga Khan University, Karachi, Pakistan,*; 3 *Department of Internal Medicine, West Virginia University, Morgantown, WV, *; 5 *Department of Leukemia, The University of Texas MD Anderson Cancer Center, Houston, TX, USA. *

**Keywords:** Primary Myelofibrosis, Thrombocytosis, Myeloproliferative Disorders

## Abstract

Primary Myelofibrosis is a BCR-ABL negative myeloproliferative neoplasm with a variety of hematological presentations, including thrombosis, bleeding diathesis and marrow fibrosis. It is estimated to have an incidence of 1.5 per 100,000 people each year. Although JAK2 or MPL mutations are seen in PMF, several other mutations have recently been documented, including mutations in CALR, epigenetic regulators like TET, ASXL1, and 13q deletions. The identification of these mutations has improved the ability to develop novel treatment options. These include JAK inhibitors like ruxolitinib, heat shock protein-90 inhibitors like ganetespib, histone deacetylase inhibitors including panobinostat, pracinostat, vorinostat and givinostat, hypomethylating agents like decitabine, hedgehog inhibitors like glasdegib, PI3K, AKT and mTOR inhibitors like everolimus as well as telomerase inhibitors like imtelstat. Research on novel therapeutic options is being actively pursued in order to expand treatment options for primary myelofibrosis however currently, there is no curative therapy other than allogenic hematopoietic stem cell transplantation (ASCT) which is possible in select patients.

## Introduction

Primary Myelofibrosis (PMF) is a classic chronic myeloproliferative neoplasm (MPN), belonging to the category of BCR-ABL negative MPNs (Hobbs and Rampal, 2015). It is estimated to have an incidence of 1.5 per 100,000 people each year, with a median age at presentation of 67 years (Mesa et al., 1999).

The clinical presentation of PMF includes hepatosplenomegaly, extramedullary hematopoiesis, expression of portal hypertension and potentially debilitating constitutional symptoms. Classical laboratory features include progressive anemia, leukocytosis, leukoerythroblastosis and thrombocytosis though leukopenia and thrombocytopenia can also occur (Scherber et al., 2011).

Currently, there is no curative therapy other than allogenic hematopoietic stem cell transplantation (ASCT). However, only a select minority of patients are eligible for this procedure (Ballen et al., 2010). Hence, the management of such patients is aimed at controlling disease progression and symptoms in order to improve quality of life for patients. 

The pathogenesis of PMF involves a variety of mutations, which include the conventional mutations responsible for the majority of diagnosed cases, as well as a multitude of other mutations implicated in a minority of patients. The 2016 World Health Organization (WHO) classification highlights this clonal nature of the disease by defining the presence of JAK2 (V617F) or MPL (W515) mutations as a major diagnostic criterion (Passamonti and Maffioli). In this review, we discuss these conventional driver mutations, other less frequently encountered mutations associated with PMF, and their role in the disease pathogenesis. We also describe novel therapeutic agents currently available for primary myelofibrosis, as well as future directions for the development of these therapies.

*Mutations encountered in PMF*

*JAK mutations*

JAK2 is a non-receptor tyrosine kinase that undergoes phosphorylation after ligand interaction. JAK2 V617F, the first driver mutation to be described, is an activating mutation in exon 14 resulting in valine-to-phenylalanine substitution (Tefferi, 2016). These mutations are seen in 50-60% of patients with PMF or essential thrombocythemia (ET) (Azzato and Bagg, 2015). The V617F mutation prevents physiologic inhibition by occupying the pseudokinase domain of JAK2, directly activating the kinase domain (Shan et al., 2014). Activation of downstream targets of this mutation, however, depends on cytokine receptor expression at various levels, particularly of homodimeric type 1 receptors (Lu et al., 2008; Vainchenker et al., 2016). This gain-of-function mutation leads to constitutive action of downstream STAT3/5 signaling. The resultant continuous JAK-STAT signaling contributes to unchecked myeloproliferation which is the hallmark of this disease (Hobbs and Rampal, 2015; Singh, 2015). 

*MPL mutations*

Another mutation reported in 11% of PMF patients involves the MPL oncogene on chromosome 1p34, which encodes the thrombopoietin receptor c-MPL (Tefferi, 2010; Singh, 2015). This type 1 cytokine receptor binds thrombopoietin and activates downstream effectors and signaling pathways including STAT3, STAT5 and PI3K/AKT – which is crucial to the survival and proliferation of megakaryocytes (Sasazawa et al., 2015).

The MPL W515L mutation occurs especially in the JAK2 V617F-negative subset of patients, and causes cytokine-independent activation of the thrombopoietin receptor (Pikman et al., 2006). Subsequent dysregulation of megakaryocyte differentiation and multiplication via overactive JAK-STAT signaling produces a myelofibrosis phenotype (Pikman et al., 2006; Defour et al., 2016). 

A similar mutation has also been described where a try-lys substitution at the same codon results in cytokine independent c-MPL receptor activation, (Pardanani et al., 2006). 

*CALR mutations*

Calreticulin gene (CALR) mutations have been implicated in PMF pathogenesis in JAK2 V617F- and MPL-negative patients (Klampfl et al., 2013). More than 50 frameshift mutations have been reported in exon 9 of CALR, all of which produce a mutant CALR protein with basic charge and loss of binding sites (Klampfl et al., 2013; Nangalia et al., 2013; Tefferi et al., 2014c; Azzato and Bagg, 2015).

The most frequent of these is del52, which is a ‘type 1’ mutation representing 55% of CALR mutations, and results in loss of all negative charge of the protein generated. On the other hand, the ‘type 2’ mutation, ins5, is seen in 30% of CALR-mutated cases and the resultant protein product retains half the negative charges of the normal protein (Klampfl et al., 2013; Vainchenker et al., 2016). Corresponding to these types of mutations, other newly discovered genetic aberrations are classified as ‘type 1’- or ‘type 2’-like (Vainchenker et al., 2016), with the former being the more common variant (75% vs 15%) (Vainchenker and Kralovics, 2017). A recent meta-analysis found the overall frequency of CALR mutations in PMF patients to be 22% (Kong et al., 2016). 

CALR is located in the endoplasmic reticulum (ER) and serves as a major component of the quality-control machinery by ensuring correct glycoprotein folding. It also contributes to calcium homeostasis, and can bind N-glycosylated residues of several proteins (Michalak et al., 2009). Preliminary studies suggest that del52 mutations activate STAT5 resulting in cytokine-independent cell growth; mutant CALR has been shown to bind and activate the MPL receptor (Araki et al., 2016; Chachoua et al., 2016). This binding and activation of MPL by mutant CALR can potentially occur at any location between the ER and cell membrane (Araki et al., 2016; Chachoua et al., 2016). Furthermore, since CALR mutants are secreted from the cell, they can also stimulate other cells such as monocytes to produce pro-inflammatory cytokines (Garbati et al., 2016). 

A recent study demonstrated that mutant CALR may be considered a MPN-specific tumor antigen, suggesting CALR to be a rational target of immunotherapy. It also showed that the limited T cell response to CALR-mutated MPN involved checkpoint signaling, and that checkpoint inhibitor therapy may be effective in these patients (Cimen Bozkus et al., 2017).

*Heterogeneous mutations seen in PMF*

An estimated 10-15% of patients with PMF do not harbor any of the 3 major mutations described above, and are so called ‘triple-negative’ (Tefferi et al., 2014b). These patients likely have a range of heterogeneous driver mutations, a large number of which have been correlated with hyperactive JAK/STAT signaling (Rampal et al., 2014). This subset of PMF patients has the poorest prognosis (Tefferi et al., 2014a). 

Certain mutations in epigenetic regulator genes have been documented in myelofibrosis, including TET2, ASXL1, EZH2, DNMT3A, and IDH1/IDH2 (Singh). Additionally, spliceosome mutations have a role in the progression of myelofibrosis (Yoshida et al., 2011; Tefferi et al., 2014a). 

TET (Ten-Eleven-Translocation) enzymes, consisting of TET1/2/3, are responsible for hydroxylating methylcytosine residues (Rampal and Levine, 2014). TET2 mutations are the most frequently occurring mutations in myelofibrosis not involving the JAK/STAT pathway (Tefferi et al., 2009). In patients harboring both aberrations, TET disruption may precede or follow JAK2 mutations (Rampal and Levine, 2014). 

Malignant myeloid diseases frequently have ASXL1 gene mutations (Carbuccia et al., 2009; Gelsi-Boyer et al., 2009). The ASXL1 protein interacts with a group of proteins called polycomb proteins, including the polycomb repressive complex 2 (PRC2) (Abdel-Wahab et al., 2012). They participate in regulating transcription via nuclear hormone receptors (Abdel-Wahab et al., 2012; Saeidi, 2016). ASXL1 mutations result in altered histone methylation, leading to increased expression of HoxA9 and HoxA10, and greater malignant potential (Abdel-Wahab et al., 2012). 

Recently, ASX has been shown to deubiquitinate histone H2A, reversing the normal function mediated by the PRC1 complex (Scheuermann et al.). The regulation of target genes such as the HOX is maintained via the balance between these 2 activities, and is essential to normal genetic expression (Sauvageau and Sauvageau, 2010). Around 19-40% of patients with PMF have ASX mutations (Singh), with CALR-negative, ASXL1-positive patients being at greatest risk of acquiring this mutation (Tefferi et al., 2014c; Singh, 2015). 

Chromosomal aberrations may also be responsible for a subset of cases of MPF. A study at the MD Anderson Cancer Center investigated genomes in PMF patients with isolated del (13q) mutations (Mehrotra et al., 2015). Compared to normal karyotype PMF, del (13q) patients had significantly higher degrees of bone marrow fibrosis and splenomegaly; however there was no difference in median overall survival between the two patient populations.

*Therapeutic targets in PMF*

**Figure 1:**
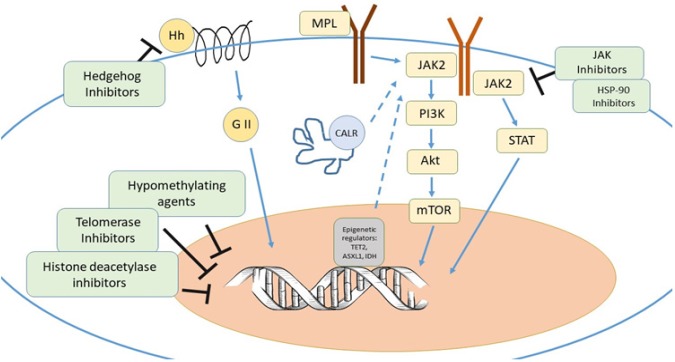
Potential Therapeutic Targets in MF are Illustrated Above

A discussion of how epigenetic mutations in PMF impact prognosis thus demonstrates the rationale for modulating epigenetic regulation. The discovery of JAK V617F and other epigenetic mutations has led to a more focused approach to the management of PMF and development of new therapies. This has the potential to reduce the burden of symptoms and morbidity and mortality associated with this disease. Some of the therapies for PMF are outlined in Figure 1 and detailed further below.

**Table 1:** Frequently Occurring Mutations in Primary Myelofibrosis

Mutation	Mutation type	Frequency	Pathophysiology
JAK2V617F	Activating mutation	50-60% of patients with ET or PMF	G to T somatic mutation resulting in valine substituting phenylalanine (Tefferi, 2016)
Thrombopoetin receptor MPL	Activating mutation	11% of patients with PMF	Mutations in exon 10 leading to MPL receptor becoming active and oncogenic (Pikman et al., 2006)
Calreticulin gene (CALR)	Abnormal protein activates MPL receptors	22% of patients with PMF	More than 50 mutations described in exon 9, with frameshift resulting in absent KDEL sequence (Klampfl et al., 2013)
Triple-negative	JAK2V617F, TpoR/MPL and CALR mutation negative	10–15% of patients with PMF or ET; Poorest prognosis	Heterogeneous, often associated with increased JAK/STAT signaling (Tefferi et al., 2014c)
ASXL1	Loss-of-function mutation of transcription regulator	19-40% of patients with PMF	Located on chromosome 20q11.1; mutations cause aberrant histone methylation (Singh, 2015)

ET, essential thrombocytopenia; PMF, primary myelofibrosis

**Table 2:** Novel Therapies Investigated in Primary Myelofibrosis

Drug	Target	Adverse effects	Study results
JAK2 Inhibitor: Ruxolitonib	JAK1 JAK2	Grade 3-4 anemia, thrombocytopenia	Primary endpoint of ≥ 35% SVR at 24 and 48 weeks seen in 42% and 28% respectively (Harrison et al., 2016)
JAK2 Inhibitor: Momelotinib (CYT387)	JAK1 JAK2	Grade 3-4 thrombocytopenia	Anemia and splenic improvement of 59% and 48 respectively. 70% of patients dependent on transfusions achieving independence for ≥12 weeks (Stein et al., 2015). Simplify 1/2 showed momelotinib falling short in comparison to ruxolitinib (O'Sullivan and Harrison, 2017)
JAK2 Inhibitor: Pacritinib (SB1518)	JAK2/FLT3 IRAK1	Gastrointestinal	PERSIST-1 showed significantly improved SVR of ≥35% at 24 weeks (19.1% of patients on pacritinib compared to 4.7% with best available therapy) (Mesa et al.). PERSIST-2 showed significantly better SVR ≥35% in the pooled pacritinib arms at 18%, vs 3% with current therapy (p=.001) (Mascarenhas et al., 2016)
HSP 90 Inhibitors: PU-H71, AUY922	JAK2, STAT3, STAT5	No clinical data currently available to report adverse effects	Preclinical data suggests Hsp90 inhibitors might improve response compared to JAK inhibition alone (Fiskus et al., 2011; Proia et al., 2011)
Rapamycin, PI3K, and AKT Inhibitors: Everolimus	(mTOR) pathway	Myelosuppression, gastrointestinal	Improved activity compared to JAK inhibition alone (Stein et al., 2015). Ruxolitinib combined with buparlisib is being evaluated (NCT01730248)
Hedgehog Inhibitor: Sonidegib	JAK-STAT hedgehog pathways	Grade 3-4 anemia, thrombocytopenia	Combination of SMO inhibitor sonidegib with ruxolitinib showed ≥ 50% decrease in splenomegaly on clinical exam in 65% of MF patients, with 9 achieving CR (Gupta et al., 2014)
HDAC Inhibitors: Panobinostat, Givinostat, Pracinostat, Vorinostat	Histone deacetylase	Grade 3-4 anemia, thrombocytopenia; GI side effects with givinostat; fatigue and cytopenias in pracinostat	Panobinostat with ruxolitinib showed >50% reduction in splenomegaly in 79% of patients, with 100% reduction in 53% (Kiladjian et al., 2014). Both drugs in combination are being currently evaluated (NCT01693601, NCT01433445, NCT02267278). (Harrison et al., 2015)
Telomerase inhibitor: Imetelstat	Telomeres	Myelosuppression, grade 3 anemia, grade 4 thrombocytopenia	Imetelstat showed complete or partial remission in 21% of primary or secondary to MF patients. Response was seen only in patients with JAK2 mutation (Tefferi et al., 2015). Two large scale trials (NCT02426086) and (NCT02598661) currently underway.
Hypomethylating agents: 5- azacytidine, decitabine	Epigenetic regulators	Grade 3-4 neutropenia, thrombocytopenia, myelosuppression	In a phase 2 trial, 5-azacytidine resulted in global hypomethylation. CR was seen only in 8 out of 34 patients and myelosupression was common (Quintas-Cardama et al., 2008). Future trials (NCT02076191) are underway.

SVR, splenic volume reduction; MF, myelofibrosis; CR, complete remission

*Janus kinase inhibitors:*

*Ruxolitinib*

Ruxolitinib is the prototype Janus kinase inhibitor drug that has demonstrated efficacy in reducing both splenic volume and MF symptoms. Ruxolitinib was approved for PMF following the results of two randomized trials showing clear splenic volume reduction (SVR). In the first trial, 41.9% of patients on ruxolitinib showed an SVR ≥ 35% compared to 0.7% in placebo at 24 weeks, while in a second trial, 28.5% of patients receiving ruxolitinib demonstrated ≥ 35% SVR at 48 weeks vs 0% with best available therapy (p < 0.0001) (Harrison et al., 2012; Verstovsek et al., 2012). Importantly, responses were noted both in patients with and without a mutated JAK V617F. Moreover, 67.0% and 79.9% of patients initially responsive to ruxolitinib therapy in both trials respectively showed long-lasting splenic responses for 48 weeks (Harrison et al., 2012; Verstovsek et al., 2012). Spleen volume reductions additionally correlated with JAK2p.V617F allele burden (Deininger et al., 2015). The most frequently reported adverse events linked to ruxolitinib were anemia and thrombocytopenia (Harrison et al., 2012; Verstovsek et al., 2012).

Final analysis results of the second trial also showed a 33% decrease in the risk of death with ruxolitinib compared to best available therapy (hazard ratio = 0.67; 95% CI, 0.44–1.02; p = 0.06). However, in a study investigating extended use of the drug noted a discontinuation rate of 92% at a median of 9.2 months. After discontinuation, severe withdrawal symptoms, labeled “ruxolitinib withdrawal syndrome” were observed. These included accelerated splenomegaly, aggravated cytopenias, and hemodynamic instability resembling septic shock (Tefferi et al., 2011).

*Momelotinib (CYT387)*

A combined phase 1/2 study of momelotinib which enrolled 163 patients (64% of whom had PMF) demonstrated reduction of splenomegaly and induction of durable anemia responses (Pardanani et al., 2011). Unlike other JAK2 inhibitors, momelotinib appeared to improve anemia, a result reproduced in another trial investigating long-term use in 60 patients. Anemia and SVR rates were 59% and 48% respectively, and 70% of patients initially requiring transfusion remained transfusion-independent for at least 12 weeks (Pardanani et al., 2013). Grade 3-4 thrombocytopenia was documented in 32% of patients. 

A recent phase 3 trial comparing momelotinib to ruxolitinib showed achievement of a primary endpoint of ≥35% SVR in 26.5% patients in the momelotinib arm versus 29% in the ruxolitinib arm at week 24, but was unable to demonstrate superiority of momelotinib in splenic size reduction (p=0.111) (O’Sullivan and Harrison, 2017). Due to these overall findings, the development of momelotinib has been aborted (O’Sullivan and Harrison, 2017).

*Pacritinib (SB1518)*

Pacritinib exerts its action by inducing JAK2/FLT3 inhibition (Hart et al., 2011), in addition to blocking IRAK1, an IL-1 receptor kinase (Singer et al., 2014). IRAK1 is overexpressed in both myelodysplastic syndromes and Fanconi anemia, which display markedly deregulated hematopoiesis (Hofmann et al., 2002; Pellagatti et al., 2010). Phase I studies showed minimal myelosuppression, and in results from a phase 2 study with 35 patients, gastrointestinal side effects were most common, notably diarrhea (Komrokji et al., 2015; Jain and Mesa, 2016). Up till week 24, 31% of patients demonstrated a ≥ 35% SVR on MRI, and 42% experienced reduction in splenic size by >50% as gauged by physical examination. Median symptom improvement was ≥50% for all symptoms except fatigue (Komrokji et al., 2015).

Regarding hematological toxicity, pacritinib appears relatively safe as compared to ruxolitinib: only 0.5% of patients receiving pacritinib experienced grade 3-4 anemia, and 4.2% experienced grade 3-4 thrombocytopenia (Beauverd et al., 2015). No statistically significant decrease in either hemoglobin levels or platelet count was evident during the treatment course when compared to baseline (Beauverd et al., 2015).

In the phase 3 study PERSIST-1, 327 patients with myelofibrosis were randomized to pacritinib or best available therapy. The primary outcome measure of an SVR of ≥35% was achieved by 19.1% patients in the pacritinib group versus 4.7% in the control group (p=0.0003) (Mesa et al., 2017). At week 60, 24% of evaluable patients receiving pacritinib maintained an SVR of ≥35%. The most frequent adverse effects included grade 3 GI symptoms such as diarrhea (5%), nausea (<1%) and vomiting (<1%) (Mesa et al., 2017). 

Subsequently, PERSIST-2 was designed to assess two different treatment regimens of pacritinib against standard therapy (Mascarenhas et al., 2016). In the final analysis, significant splenic response (≥35% SVR) was seen in the pooled pacritinib arms compared to standard therapy (18% in pooled pacritinib arms versus 3% with standard therapy, p=.001); however, improved symptom control as determined by significant reduction in total symptom score (by ≥50%) was not observed with pacritinib therapy (Mascarenhas et al., 2016).

Significant cardiovascular and hemorrhagic events occurred in both PERSIST trials. In particular, increased mortality from cardiac failure, cardiac arrest and intracerebral hemorrhage was seen in the pacritinib arm of PERSIST-1. This led to the FDA halting the drug in February 2016 (Bose et al., 2017). However the ban was lifted in January 2017 following submission of final clinical study reports, and pacritinib is currently under study in another clinical trial (NCT03165734) (Bose et al., 2017).

*The challenges of JAK inhibition*

Although JAK inhibitors constitute the basis of treatment for myelofibrosis, there are certain issues associated with their use. For one, myelosuppression, although manageable, is a common toxicity. Secondly, JAK inhibitors are unable to eradicate the mutant JAK. Furthermore, they do not selectively target mutant JAK2 alone, potentially leading to adverse effects including cytopenias that result from disruption of normal JAK signaling (Stein et al., 2015). Impaired dendritic cell function resulting from JAK1/JAK 2 inhibition can increase susceptibility to infection, while blockade of FLT3 can cause diarrhea (Heine et al., 2013).

In the long-term, JAK/STAT activation can persist despite the use of JAK inhibitors via the formation of heterogenous dimers with other JAK proteins (Koppikar et al., 2012). Finally, the pathogenesis of PMF involves molecular abnormalities at multiple levels which may not all be targeted by a single agent (Stein et al., 2015). This was demonstrated in an* in vivo* study which showed that resistance to JAK inhibitors can arise via the activation of PDGF-mediated MAPK signaling pathways independent of JAK blockade (Meyer et al., 2017). Further data also suggests that modulating this pathway through the use of MEK inhibitors decreases the activity of MAPK and its downstream pathways (Meyer et al., 2017). This allows for another potential treatment modality for use in PMF. 

Other agents which can be used for treatment of MF either alone or in combination with JAK inhibitors are discussed below. 

*Eliminating resistance to JAK inhibition – HSP-90 inhibitors*

Heat shock proteins (Hsp) represent a group of chaperone molecules tasked with facilitating correct protein folding. In particular, Hsp-90 mediates folding of approximately 200 proteins, many of which are involved in normal cellular signaling (Jhaveri and Modi, 2012). PU-H71 is an Hsp-90 inhibitor that regulates JAK2 expression and thus reduces activation of downstream pathways including STAT3/5, resulting in cell death in both MPL and JAK-mutant clones (Santos and Verstovsek, 2013). In a separate study, another HSP-90 inhibitor, AUY922 decreased mutant JAK expression and increased apoptosis of CD34+ cells (Fiskus et al., 2011; Stein et al., 2015). Ganetespib, another HSP-90 inhibitor, decreased in vitro and in vivo STAT activity (Proia et al., 2011). Hsp-90 inhibition thus may be a potential strategy for overcoming ruxolitinib resistance mechanisms, although at present clinical data is limited.

*Addressing epigenetic regulation*

*Histone deacetylase inhibitors*

Panobinostat, pracinostat, vorinostat and givinostat are four drugs in this class that have been investigated in patients with primary myelofibrosis, with the most encouraging preliminary results coming from panobinostat (Mascarenhas et al., 2013; Stein et al., 2015). A phase 1 study showed clinical response in 5 out of 18 PMF patients, of whom 3 had a 100% reduction in splenomegaly, and 2 had improvement in anemia (Mascarenhas et al., 2017). Panobinostat along with ruxolitinib was also studied in 61 MF patients. An SVR >50% was achieved in 79% of patients, with 53% having a non-palpable spleen on physical exam (Kiladjian et al., 2014). Adverse events were similar to ruxolitinib monotherapy, i.e. anemia and thrombocytopenia. 

In preclinical studies, ruxolitinib and panobinostat showed synergistic activity, with an improvement in fibrosis and reduced bone marrow cellularity (Baffert et al., 2011; Stein et al., 2015). As a result, this combination is now being evaluated in 3 clinical trials (NCT01693601, NCT01433445, and NCT02267278). Preliminary results from one study with 23 patients (NCT01433445) reported a ≥35% SVR in 57% and 39% of patients at 24 and 48 weeks respectively, as well as improvement in bone marrow fibrosis in 4 patients. A ≥20% decrease in the JAK2 V617F allele burden was seen in 5 patients at 48 weeks (Harrison et al., 2015). These results show a better outcome than that expected with ruxolitinib alone, and detailed results are awaited. 

In individual phase 2 studies, other histone deacetylase inhibitors tended to have a moderate to low response, and demonstrated adverse effects which restricted their use. Givinostat induced gastrointestinal side effects in 62% of patients; while several patients on pracinostat experienced fatigue and cytopenias (Rambaldi et al., 2010). 

*Hypomethylating agent*s 

ASX1, an epigenetic regulator of MF, is susceptible to hypomethylation. 5-azacytidine and decitabine are two such hypomethylating agents that have been studied in patients with MF. A phase 2 trial with 34 patients given 5-azacytidine showed hypomethylation in all patients, but clinical improvement was recorded in only 8 patients, and myelosupression was commonly observed (Quintas-Cardama et al., 2008). Similarly, in 21 patients with myelofibrosis treated with decitabine, a response was seen in 7 of 19 evaluable patients; reduction in spleen size was not reported (Odenike et al., 2008). Grade 3/4 neutropenia and thrombocytopenia was seen in 95% and 52% of patients in this cohort (Odenike et al., 2008). Decitabine in combination with ruxolitinib is under investigation at present (NCT02076191).

*Drugs targeting other signaling pathways*

*Hedgehog inhibitors*

Hedgehog proteins are lipid-modified signaling proteins with a final downstream anti-apoptotic effect (Sochacki et al., 2016), and have been shown to interact in MF. Preclinical models showed that granulocytes derived from MPN patients had increased hedgehog target gene expression compared to controls. Murine models treated with ruxolitinib in combination with a smoothened (SMO) inhibitor had lower mutant-allele burden than those treated with either drug alone. Bone marrow fibrosis was also reduced (Keller et al., 2013). In a Phase 1 trial with 23 patients, combination therapy with sonidegib (LDE226), an SMO inhibitor, and ruxolitinib was investigated (Gupta et al., 2014). 65% of MF patients had a ≥ 50% reduction in splenomegaly on physical exam, with an impalpable spleen in 9 patients. Anemia and thrombocytopenia were documented in a minority of patients (Gupta et al., 2014). 

Glasdegib, another SMO inhibitor is being investigated currently in a Phase 1b/2 trial of 21 patients with MF and preliminary results suggest modest clinical activity of this drug. 5 patients showed a spleen volume reduction whilst one patient had improvement in anemia. It should be noted that 52% of patients (n=11) were refractory to treatment (and had prior JAK inhibitor treatment). Dysgeusia and muscle spasms were frequent side effects (Gerds et al., 2017). 

*Inhibiting PI3K, AKT and mTOR pathways*

The PI3K/AKT/mTOR pathway functions downstream of JAK/STAT, and thus can serve as a potential target for therapeutic inhibition in MF. This pathway is active in myeloproliferative neoplasms, and acts by dysregulating cell death (Guglielmelli et al., 2011). Everolimus, an mTOR inhibitor, was investigated in a study of 30 patients with myelofibrosis. Clinical improvement was seen in six patients (reduction in spleen volume in 5 patients, raised hemoglobin in 1 patient), while one patient even had a partial remission (Guglielmelli et al., 2011). 

Drugs from this class have also been shown to act synergistically when combined with JAK inhibitors (Vannucchi et al., 2011; Bogani et al., 2013). Ruxolitinib in combination with buparlisib (BKM120) has been investigated in a phase 1b trial (NCT01730248), and the results are expected to be made available soon. The AKT inhibitor MK-2206 demonstrated decreased hepatosplenomegaly and megakaryocyte burden in animal models, and decreased megakaryocyte colony formation in patient samples (Khan et al., 2013). When used in conjunction with ruxolitinib, synergistic inhibition of cell growth in JAK mutants was seen, warranting clinical investigation.

*Telomerase inhibition: An additional novel strategy*

Repetitive telomere sequences found at the end of chromosomes function to protect coding DNA from genetic damage (Cerquozzi et al., 2016). As cells age, the telomere sequences at the end of their chromosomes become shorter, eventually leading to programmed cell death. There is dramatic upregulation of the telomerase enzyme in malignant cells, conferring unlimited replication potential. This makes the telomerase enzyme a relatively specific target for therapy (Sochacki et al., 2016). The telomerase inhibitor imetelstat was administered to 33 primary/secondary MF patients in a pilot study (Tefferi et al., 2015). 21% of these patients demonstrated a response, and 4 patients achieved complete remission with bone marrow fibrosis reversal, with 3 of these additionally demonstrating a molecular response. Interestingly, responses were seen only in patients with JAK2 mutation, whereas ASXL1-mutant patients were unaffected. Myelosuppression was the most significant adverse event reported along with grade 3 anemia and grade 4 thrombocytopenia (Tefferi et al., 2015).

Overall, there is significant potential in telomerase inhibitor therapy. Two large scale trials, IMbark (NCT02426086) and IMerge (NCT02598661), studying imtelsat are ongoing at present. 

*Future perspective*

Despite the multitude of clinical trials investigating various therapies for PMF currently underway, there remains a dearth of information about disease pathogenesis. Further work is needed to elucidate mechanisms of disease initiation, as JAK2 V617F is a mutation that is frequently acquired but does not always lead to disease. Likewise it also remains to be understood why JAK2 V617F and mutant CALR pathways cause distinct (albeit closely related) diseases, even though both activate the same MPL/JAK2 pathway.

Additional gene mutations besides JAK2 V617F are thought to be involved in the pathophysiology of myeloproliferative neoplasms, and additional studies are needed to confirm the pathogenetic role of other phenotypic modifiers such as epigenetic regulators.

As recommended by the 2016 WHO classification of myeloproliferative neoplasms, further research is required to fully characterize the influence of molecular findings on disease prognosis. It is particularly relevant in the context of pre-myelofibrosis to determine the impact of CALR/ASXL1 status on prognosis (Passamonti and Maffioli, 2016). 

Given the multifactorial nature of the disease, with many interlinked genetic and environmental elements implicated in its pathophysiology, it remains to be seen which multi-drug regimen will serve as the best treatment of choice for primary myelofibrosis. Hypomethylating agents are a group of epigenetic regulators that may be a promising class of drugs for primary myelofibrosis, especially in combination with ruxolitinib. NS-018 is a JAK inhibitor currently in development, and has shown encouraging results that merit its further investigation. Interferon has also been proposed as a therapeutic option for PMF, but data from this patient population is limited.

Lastly, as discussed in this review, PI3K/mTOR/AKT inhibitors and telomerase inhibitors have also shown much potential, and further studies are warranted to explore this treatment option for primary myelofibrosis. 

In conclusion, our understanding of PMF pathogenesis has improved vastly in the past decade, due in major part to the discovery of some key driver mutations that underlie this disease. Recent genomic studies have shown that PMF can progress to a more advanced form of myeloproliferative neoplasia (MPN): a heterogenous disorder that exhibits phenotypic and genotypic features of both myeloproliferative and myelodysplastic syndrome (MDS), called MPN/MDS. Due to this, monotherapy appears to be a suboptimal strategy, thus warranting the development of novel combination approaches for patients with primary myelofibrosis. Several of the new therapeutic options discussed here have shown some promise in early trials, and are expected to be investigated more thoroughly for their potential to reduce the morbidity and mortality associated with this disease.

*Executive summary*

• Primary myelofibrosis is a myeloproliferative neoplasm that has a diverse range of underlying genetic causes.

• Genetic mutations in JAK, MPL and CALR genes are common drivers of this disease.

• Mutations in epigenetic regulators such as ASXL1, and spliceosome components, as well as TET enzymes have also been shown to cause disease.

• Some patients, described as “triple negative”, do not express any of the 3 major mutations; this subset has the poorest prognosis. 

• Allogenic stem cell transplant (ASCT) remains the only definitive cure, but this procedure can only be performed in a selected patients. The associated high morbidity and mortality further points out the urgent need to develop alternative therapeutic modalities. 

• JAK2 inhibitors, in particular ruxolitinib, are used to treat PMF and produce significant reduction in splenomegaly, but are associated with significant anemia and thrombocytopenia. 

• Significant inherent challenges associated with JAK inhibitor monotherapy warrant the development of new drug modalities and multi-drug regimens.

• Other therapeutic modalities that can potentially be used in conjunction with JAK2 inhibitors include epigenetic modulators, hedgehog inhibitors, inhibitors of PI3K/AKT/mTOR pathways, and telomerase inhibitors.

• Epigenetic regulator mutations, the target of histone deacetylase inhibitors, have shown significant reduction in splenomegaly when used in combination with JAK inhibitors. Hypomethylating agents however, have limited clinical response.

• The SMO inhibitor sonidegib when used in conjunction with ruxolitinib showed a significant reduction in splenomegaly in approximately two-thirds of patients with PMF in a phase I study.

• PI3K/mTOR inhibitors and JAK1/2 inhibitors in combination have caused synergistic inhibition of myeloproliferative neoplastic cells. 

• Lastly, telomerase inhibitors such as imtelstat have shown encouraging results in pilot studies, though their efficacy is limited to patients with JAK2 mutations alone. 
